# Impact of treatment interruption on the efficacy and safety of vunakizumab in patients with moderate-to-severe plaque psoriasis: a *post-hoc* analysis of a phase 3 trial

**DOI:** 10.3389/fimmu.2025.1639049

**Published:** 2026-01-16

**Authors:** Qunyan Li, Weifeng Yao, Dongyun Lei, Yan Xu, Litao Zhang, Zuotao Zhao, Junchen He, Tao Guo, Junying Li

**Affiliations:** 1 Graduate School, Tianjin Medical University, Tianjin, China; 2 Department of Dermatology, Tianjin Academy of Traditional Chinese Medicine Affiliated Hospital, Tianjin, China; 3 Department of Dermatology, Tianjin lnstitute of lntegrative Dermatology, Tianjin Academy of Traditional Chinese Medicine Affiliated Hospital, Tianjin, China

**Keywords:** moderate-to-severe plaque psoriasis, vunakizumab, treatment interruption, efficacy, safety

## Abstract

**Objective:**

Vunakizumab, a novel IL-17A inhibitor, has demonstrated satisfactory efficacy and safety for the treatment of moderate-to-severe plaque psoriasis. This analysis aimed to assess the impact of treatment interruption on the efficacy and safety of vunakizumab in the treatment of this disease.

**Methods:**

This *post-hoc* analysis used data from a phase 3 trial of vunakizumab (NCT04839016) that enrolled patients with moderate-to-severe plaque psoriasis. A total of 460 patients received vunakizumab treatment and were included in this analysis.

**Results:**

Over the 52-week treatment, 223 patients had one or more treatment interruption, and 237 patients had no treatment interruption. At week 52, patients with treatment interruption had lower achievement rates for Psoriasis Area and Severity Index (PASI) 75 (77.1% vs. 97.9%), PASI 90 (67.3% vs. 94.1%), PASI 100 (49.8% vs. 75.9%), and static Physician’s Global Assessment of 0/1 (62.8% vs. 93.7%) than those without interruption (all *P*<0.001). Additionally, at week 52, patients with treatment interruption had lower improvements in patient-reported outcomes (PROs), including Dermatology Life Quality Index score, Itch Numerical Rating Scale score, EuroQol-5D and visual analogue scale score, and Short Form-36 Mental Component Score than those without interruption (all *P*<0.05). Further subgroup analysis indicated that the increased frequency of treatment interruption correlated with poorer PASI responses and PROs (all *P*<0.05). The incidence of overall adverse events was similar between the two groups.

**Conclusion:**

Interrupted vunakizumab treatment reduced the clinical response and quality of life in patients with moderate-to-severe plaque psoriasis.

## 1 Introduction

Plaque psoriasis is the most common form of psoriasis, accounting for more than 80% of psoriasis cases (1). Over the last 20 years, biologics have greatly revolutionized the treatment landscape of plaque psoriasis, leading to improved treatment response and quality of life in patients with plaque psoriasis (2–6). However, biological treatment interruption occurs in some patients with plaque psoriasis (7), and interrupting these treatments is responsible for reduced efficacy (8–10). Therefore, continuously administering biologics is crucial in improving the clinical outcomes of patients with plaque psoriasis.

Among the therapeutic targets of biological agents, interleukin (IL)-17A plays a pivotal role in the pathogenesis of plaque psoriasis (11–13). IL-17A interacts with its receptor to stimulate the production of cytokines and chemokines, which further promotes hyperproliferation and alters the differentiation of keratinocytes, thereby facilitating psoriasis (11, 14, 15). Thus, inhibition of IL-17A can ameliorate psoriasis.

Vunakizumab (SHR-1314), a novel IL-17A inhibitor, has been approved for the treatment of moderate-to-severe plaque psoriasis in China (16). According to the phase 3 trial (NCT04839016), vunakizumab treatment resulted in satisfactory efficacy during 52 weeks compared to placebo with good tolerability in patients with moderate-to-severe plaque psoriasis (17). However, the impact of treatment interruption on the efficacy and safety of vunakizumab in patients with moderate-to-severe plaque psoriasis is unclear.

This *post-hoc* analysis extracted data from the phase 3 trial (NCT04839016) and aimed to investigate the effect of vunakizumab treatment interruption on treatment responses, patient-reported outcomes (PROs), and adverse events in patients with moderate-to-severe plaque psoriasis.

## 2 Methods

### 2.1 Study design and population

This was a *post-hoc* analysis of a randomized, double-blind, parallel, placebo-controlled, multicenter phase 3 trial (NCT04839016). In brief, 460 patients with moderate-to-severe plaque psoriasis who were treated with 52-week vunakizumab were selected. The ‘moderate-to-severe’ was defined as a psoriasis area and severity index (PASI) score of 12 or higher, a static physician’s global assessment (sPGA) score of 3 or higher, and equal to or more than 10% body surface area (BSA) affected by psoriasis. The objective of this *post-hoc* analysis was to assess the impact of treatment interruption on the efficacy and safety of vunakizumab in patients with moderate-to-severe plaque psoriasis. The detailed inclusion and exclusion criteria for patients with moderate-to-severe plaque psoriasis were published in the previous phase 3 trial (17). This study received ethical approval from the institutional review boards at each center.

### 2.2 Treatment interruption

Treatment interruption was defined as one or more times of vunakizumab discontinuation at any time point over the 52-week treatment period, regardless of the reason. Based on this definition, patients with moderate-to-severe plaque psoriasis who had one or more times of vunakizumab discontinuation were categorized into the treatment interruption group (N=223), while patients in the continuous treatment group were those without discontinuation of vunakizumab treatment (N=237).

### 2.3 Outcomes

This *post-hoc* analysis contained the following outcomes: (1) long-term responses at the 52^nd^ week after treatment initiation (W52), including PASI 75, PASI 90, PASI 100, and sPGA 0/1 responses; (2) PROs at W52, including the Dermatology Life Quality Index (DLQI) score (18), DLQI 0/1 response rate, Itch Numerical Rating Scale (I-NRS) (19), EuroQol-5D (EQ-5D) utility index, EQ-5D and visual analogic scale (VAS) (Available versions | EuroQol), and Short Form-36 (SF-36) Mental Component Score (MCS) and Physical Component Score (PCS) (20); (3) adverse events.

### 2.4 Subgroup analysis

To investigate how different times of treatment interruption affect the efficacy and safety of vunakizumab, patients were categorized into four subgroups based on the times of treatment interruption: 0 (n=237), 1 (n=62), 2 (n=67), and ≥3 times (n=94). The outcomes involving long-term response, PROs, and adverse events were compared among four subgroups. Furthermore, the median duration of drug exposure was 364 days. Based on this, the long-term response was further compared between patients with short (<median duration) and lengthy (≥median duration) drug exposure.

### 2.5 Statistical analysis

The analyzed data were collected from the phase 3 trial (NCT04839016) (17). This *post-hoc* analysis was performed from August to November 2024. SPSS 29.0 (IBM, USA) was used for statistical analysis with a *P*<0.05 indicating statistical significance. All analyses were *post-hoc* and not adjusted for multiplicity. The comparison of long-term response was conducted via the *χ*^2^ test between groups or among four subgroups. The comparison of continuous variables in PROs was analyzed via student *t-*test between groups and ANOVA among four subgroups, while the comparison of categorical variables in PROs was analyzed via *χ*^2^ test. The comparison of adverse events between groups and among four subgroups was performed via *χ*^2^ test or Fisher’s exact test. Following adjustment for baseline variables, outcomes between the treatment interruption and continuous treatment groups were compared using analysis of covariance (ANCOVA) or multivariate logistic regression analysis.

## 3 Results

### 3.1 Baseline characteristics of the patients in the treatment interruption and continuous treatment groups

Age (*P*=0.684) and sex (*P*=0.423) did not differ between the two groups. However, family history of psoriasis (*P*=0.003), hyperlipemia (*P*=0.035), type II diabetes mellitus (DM) (*P*=0.021), PASI score (*P*=0.028), sPGA score (*P*=0.036), and SF-36 PCS (*P*=0.008) were different between the two groups. Other clinical features were not different between the two groups, including race, body mass index, smoking status, drinking status, DM, hypertension, hyperuricemia, disease duration, BSA score, DLQI score, I-NRS score, EQ-5D utility index score, EQ-5D VAS score, and SF-36 MCS (all *P*>0.05) (**Table 1**).

**Table 1:** Clinical characteristics of patients with moderate-to-severe plaque psoriasis.

Characteristics	Continuous treatment (N=237)	Treatment interruption (N=223)	*P* value
Age, n (%)			0.684
<65 years	221 (93.2)	210 (94.2)	
≥65 years	16 (6.8)	13 (5.8)	
Sex, n (%)			0.423
Female	52 (21.9)	56 (25.1)	
Male	185 (78.1)	167 (74.9)	
Race, n (%)			
Asian	237 (100.0)	223 (100.0)	(-)
BMI (kg/m^2^), mean ± SD	25.1 ± 3.5	25.6 ± 4.3	0.146
Smoking, n (%)			0.677
Never	130 (54.9)	118 (52.9)	
Former or current	107 (45.1)	105 (47.1)	
Drinking, n (%)			
No	237 (100.0)	223 (100.0)	(-)
Family history of PsO, n (%)			0.003
No	202 (85.2)	165 (74.0)	
Yes	35 (14.8)	58 (26.0)	
Hypertension, n (%)			0.325
No	195 (82.3)	191 (85.7)	
Yes	42 (17.7)	32 (14.3)	
Hyperlipemia, n (%)			0.035
No	190 (80.2)	195 (87.4)	
Yes	47 (19.8)	28 (12.6)	
DM, n (%)			0.061
No	209 (88.2)	208 (93.3)	
Yes	28 (11.8)	15 (6.7)	
Type II DM, n (%)			0.021
No	213 (89.9)	213 (98.2)	
Yes	24 (10.1)	10 (4.5)	
Hyperuricemia, n (%)			0.170
No	205 (86.5)	202 (90.6)	
Yes	32 (13.5)	21 (9.4)	
Disease duration (years), mean ± SD	11.6 ± 9.8	11.7 ± 9.1	0.912
PASI score, mean ± SD	23.1 ± 9.4	21.3 ± 8.3	0.028
sPGA score, n (%)			0.036
3	108 (45.6)	80 (35.9)	
4	110 (46.4)	120 (53.8)	
5	19 (8.0)	23 (10.3)	
BSA (%), mean ± SD	35.6 ± 17.4	33.3 ± 17.2	0.081
DLQI score, mean ± SD	10.7 ± 6.6	11.8 ± 7.3	0.102
I-NRS score, mean ± SD	5.6 ± 2.4	5.6 ± 2.8	0.737
EQ-5D utility index, mean ± SD	0.9 ± 0.1	0.9 ± 0.2	0.327
EQ-5D VAS score, mean ± SD	80.7 ± 16.1	80.0 ± 16.5	0.640
SF-36 MCS, mean ± SD	48.3 ± 10.0	47.3 ± 10.5	0.292
SF-36 PCS, mean ± SD	52.2 ± 5.7	50.6 ± 6.8	0.008

BMI, body mass index; SD, standard deviation; PsO, psoriasis; DM, diabetes mellitus; PASI, psoriasis area and severity index; sPGA, static physician’s global assessment; BSA, body surface area; DLQI, dermatology life quality index; I-NRS, itch numerical rating scale; EQ-5D VAS, EuroQol-5D and visual analogic scale; SF-36, short form-36; MCS, mental component score; PCS, physical component score.

### 3.2 Comparison of treatment responses at W52 between treatment interruption and continuous treatment groups

**Figure 1:**
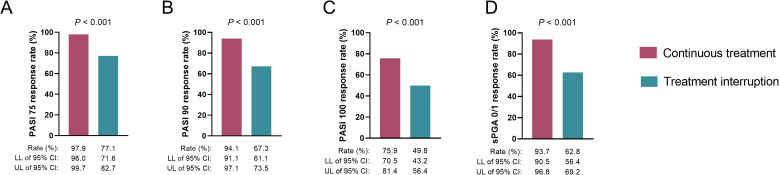
Association between vunakizumab treatment interruption and treatment responses at W52 in patients with moderate-to-severe plaque psoriasis. Association of vunakizumab treatment interruption with PASI 75 **(A)**, PASI 90 **(B)**, PASI 100 **(C)**, and sPGA 0/1 **(D)** responses at W52 in patients with moderate-to-severe plaque psoriasis.

At W52, achievement rates for PASI 75 (77.1% vs. 97.9%) (*P*<0.001) (**Figure 1A**), PASI 90 (67.3% vs. 94.1%) (*P*<0.001) (**Figure 1B**), PASI 100 (49.8% vs. 75.9%) (*P*<0.001) (**Figure 1C**), and sPGA 0/1 (62.8% vs. 93.7%) (*P*<0.001) (**Figure 1D**) responses were lower in the treatment interruption group than in the continuous treatment group.

### 3.3 Subgroup analysis for treatment responses

At W52, achievement rates for PASI 75 (*P*<0.001), PASI 90 (*P*<0.001), PASI 100 (*P*<0.001), and sPGA 0/1 (*P*<0.001) responses were the highest in patients without treatment interruption, followed by patients with 1 time and 2 times of treatment interruption, and the lowest in patients with ≥3 times of treatment interruption (**Table 2**). Subgroup analysis based on drug exposure duration showed that achievement rates for PASI 75/90/100 and sPGA 0/1 responses were higher in patients with lengthy drug exposure than those with short drug exposure (all *P*<0.05) (**Supplementary Table 1**).

**Table 2:** Comparison of long-term response among patients with different times of treatment interruption.

Items	Times of treatment interruption				*P* value
	0 (n=237)	1 (n=62)	2 (n=67)	≥3 (n=94)	
PASI 75 response, n (%)					<0.001
No	5 (2.1)	6 (9.7)	10 (14.9)	35 (37.2)	
Yes	232 (97.9)	56 (90.3)	57 (85.1)	59 (62.8)	
PASI 90 response, n (%)					<0.001
No	14 (5.9)	9 (14.5)	20 (29.9)	44 (46.8)	
Yes	223 (94.1)	53 (85.5)	47 (70.1)	50 (53.2)	
PASI 100 response, n (%)					<0.001
No	57 (24.1)	22 (35.5)	31 (46.3)	59 (62.8)	
Yes	180 (75.9)	40 (64.5)	36 (53.7)	35 (37.2)	
sPGA 0/1 response, n (%)					<0.001
No	15 (6.3)	13 (21.0)	21 (31.3)	49 (52.1)	
Yes	222 (93.7)	49 (79.0)	46 (68.7)	45 (47.9)	

PASI, psoriasis area and severity index; sPGA, static physician’s global assessment.

### 3.4 Comparison of PROs at W52 between treatment interruption and continuous treatment groups

**Figure 2:**
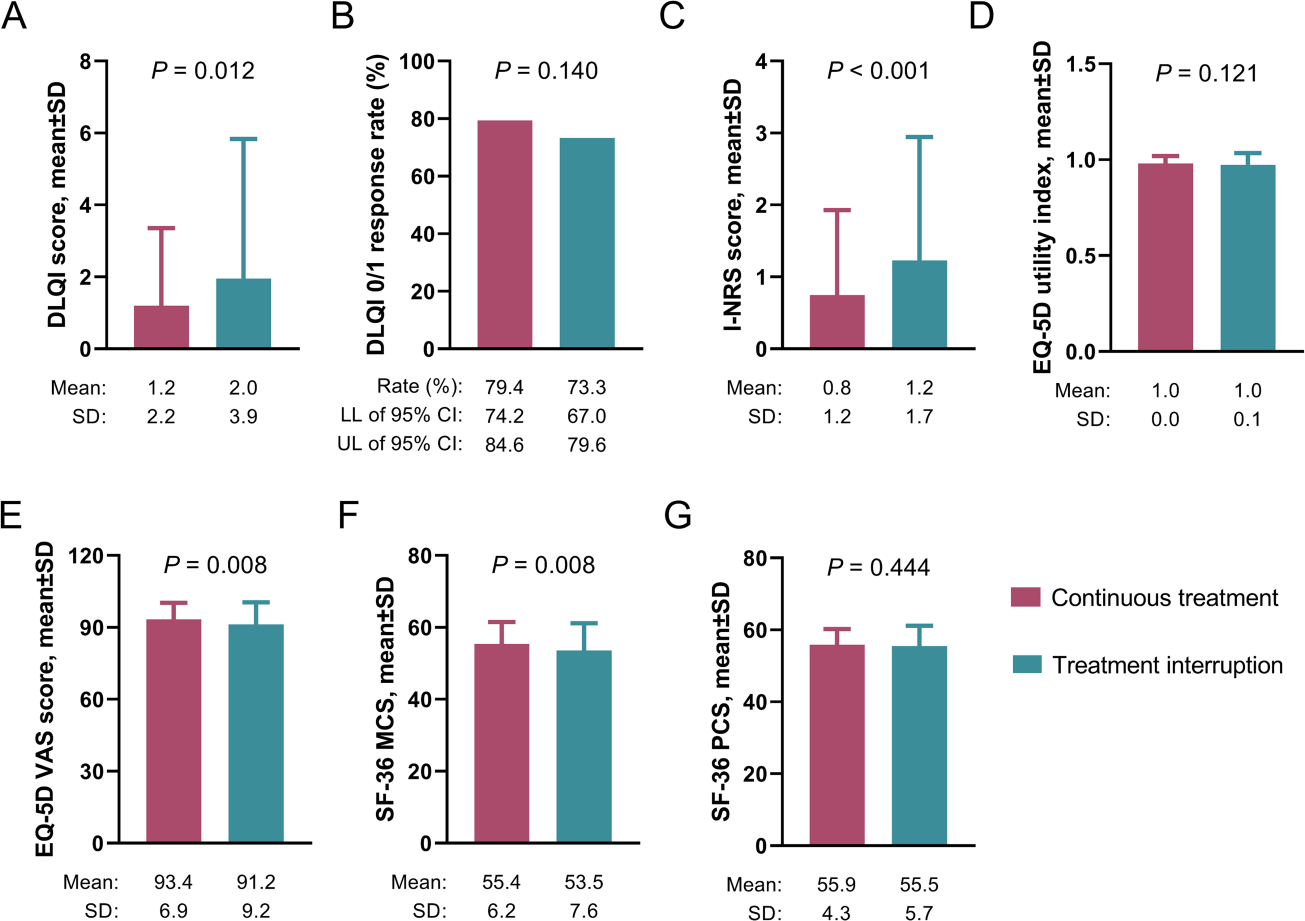
Association between vunakizumab treatment interruption and PROs at W52 in patients with moderate-to-severe plaque psoriasis. Association of vunakizumab treatment interruption with DLQI score **(A)**, DLQI 0/1 response **(B)**, I-NRS score **(C)**, EQ-5D utility index score **(D)**, EQ-5D VAS score **(E)**, SF-36 MCS **(F)**, and SF-36 PCS **(G)** in patients with moderate-to-severe plaque psoriasis.

At W52, the mean DLQI score was higher in the treatment interruption group than in the continuous treatment group (2.0 ± 3.9 vs. 1.2 ± 2.2) (*P*=0.012) (**Figure 2A**). However, achievement rates for DLQI 0/1 did not differ between the two groups (73.3% vs. 79.4%) (*P*=0.140) (**Figure 2B**). Similarly, the mean I-NRS score was higher in the treatment interruption group than in the continuous treatment group (1.2 ± 1.7 vs. 0.8 ± 1.2) (*P*<0.001) (**Figure 2C**). The mean EQ-5D utility index score did not differ between the two groups (1.0 ± 0.1 vs. 1.0 ± 0.0) (*P*=0.121) (**Figure 2D**). Additionally, the mean EQ-5D VAS score (91.2 ± 9.2 vs. 93.4 ± 6.9) (*P*=0.008) (**Figure 2E**) and SF-36 MCS (53.5 ± 7.6 vs. 55.4 ± 6.2) (*P*=0.008) (**Figure 2F**) were lower in the treatment interruption group than in the continuous treatment group. Nevertheless, the mean SF-36 PCS was not different between the two groups (55.5 ± 5.7 vs. 55.9 ± 4.3) (*P*=0.444) (**Figure 2G**).

### 3.5 Subgroup analysis for PROs

At W52, the mean DLQI score showed an increasing trend with the increase in times of treatment interruption (*P*<0.001), and achievement rates for DLQI 0/1 showed a decreasing trend (*P*=0.007). The mean EQ-5D utility index score (*P*=0.037), EQ-5D VAS score (*P*<0.001), and SF-36 MCS (*P*=0.002) and PCS (*P*=0.003) at W52 gradually decreased with the increment in times of treatment interruption (**Table 3**).

**Table 3:** Comparison of PROs among patients with different times of treatment interruption.

Items	Times of treatment interruption				*P* value
	0 (n=237)	1 (n=62)	2 (n=67)	≥3 (n=94)	
DLQI score, mean ± SD	1.2 ± 2.2	0.9 ± 1.6	1.5 ± 2.7	3.2 ± 5.4	<0.001
DLQI 0/1 response, n (%)					0.007
No	48 (20.6)	8 (13.6)	16 (27.1)	27 (37.0)	
Yes	185 (79.4)	51 (86.4)	43 (72.9)	46 (63.0)	
I-NRS score, mean ± SD	0.8 ± 1.2	0.7 ± 1.0	1.3 ± 1.8	1.6 ± 2.0	<0.001
EQ-5D utility index, mean ± SD	1.0 ± 0.0	1.0 ± 0.0	1.0 ± 0.1	1.0 ± 0.1	0.037
EQ-5D VAS score, mean ± SD	93.4 ± 6.9	93.1 ± 8.1	92.7 ± 7.5	88.5 ± 10.7	<0.001
SF-36 MCS, mean ± SD	55.4 ± 6.2	54.8 ± 6.2	54.3 ± 8.5	51.9 ± 7.7	0.002
SF-36 PCS, mean ± SD	55.9 ± 4.3	56.4 ± 5.0	56.6 ± 4.9	53.8 ± 6.5	0.003

PROs, patient-reported outcomes; DLQI, dermatology life quality index; I-NRS, itch numerical rating scale; EQ-5D VAS, EuroQol-5D and visual analogic scale; SF-36, short form-36; MCS, mental component score; PCS, physical component score.

### 3.6 Comparison of adverse events between treatment interruption and continuous treatment groups

The incidence of any adverse events was not different between the two groups (88.2% vs. 87.4%) (*P*=0.808). However, the incidence of hyperuricemia (13.5% vs. 24.1%) (*P*=0.004) and hyperlipidemia (9.9% vs. 16.5%) (*P*=0.037) was lower in the treatment interruption group than in the continuous treatment group. The incidence of elevated blood glucose (14.3% vs. 3.4%) (*P*<0.001) was higher in the treatment interruption group than in the continuous treatment group (**Table 4**).

**Table 4:** Adverse events.

Events, n (%)	Continuous treatment (N=237)	Treatment interruption (N=223)	*P* value
Any	209 (88.2)	195 (87.4)	0.808
Hyperuricemia	57 (24.1)	30 (13.5)	0.004
URTI	47 (19.8)	54 (24.2)	0.256
Hyperlipidemia	39 (16.5)	22 (9.9)	0.037
Injection site reaction	23 (9.7)	27 (12.1)	0.408
Elevated ALT	20 (8.4)	31 (13.9)	0.062
Eczema	17 (7.2)	10 (4.5)	0.220
Elevated blood bilirubin	16 (6.8)	11 (4.9)	0.407
Pruritus	15 (6.3)	11 (4.9)	0.517
Urticaria	11 (4.6)	17 (7.6)	0.181
Elevated AST	10 (4.2)	14 (6.3)	0.321
Elevated blood glucose	8 (3.4)	32 (14.3)	<0.001

URTI, upper respiratory tract infection; ALT, alanine aminotransferase; AST, aspartate aminotransferase.

### 3.7 Subgroup analysis for adverse events

The incidence of any adverse events was not affected by different times of treatment interruption (*P*=0.376). The incidence of hyperuricemia, hyperlipidemia, elevated aspartate aminotransferase (AST), and elevated blood glucose differed by different times of treatment interruption (all *P*<0.05) (**Supplementary Table 2**).

### 3.8 Comparisons of efficacy and safety between treatment interruption and continuous treatment groups after adjusting for baseline variables

Regarding treatment response, treatment interruption (vs. continuous treatment) was independently related to a lower probability of achieving PASI 75, PASI 90, PASI 100, and sPGA 0/1 responses at W52 (all *P*<0.001). Regarding PROs, treatment interruption (vs. continuous treatment) was independently related to a higher I-NRS score at W52 (*P*=0.026). Regarding adverse events, treatment interruption (vs. continuous treatment) was not related to any adverse events (*P*=0.197) (**Supplementary Table 3**).

### 3.9 Factors contributing to treatment interruption

The Coronavirus Disease-19 (COVID-19) epidemic was the major reason for treatment interruption. Specifically, 90.3%, 94.0%, and 84.0% of patients experienced 1, 2, and ≥3 times of treatment interruption, respectively, due to the COVID-19 epidemic. Additionally, 6.5%, 10.4%, and 24.5% of patients experienced 1, 2, and ≥3 times of treatment interruption, respectively, due to adverse events. A total of 11.3%, 22.4%, and 16.0% of patients experienced 1, 2, and ≥3 times of treatment interruption, respectively, due to other reasons (**Supplementary Table 4**).

## 4 Discussion

Recently, several studies have focused on the impact of biological treatment interruption on treatment response in patients with moderate-to-severe plaque psoriasis (8–10, 21, 22). As reported by a previous study, at W52, achievement rates for sPGA 0/1, PASI 75, PASI 90, and PASI 100 responses were lower in patients with moderate-to-severe plaque psoriasis with risankizumab treatment interruption than those without interruption (8). Another study reported that achievement rates for Investigator Global Assessment score of 0 (IGA 0), IGA 0/1, PASI 75, PASI 90, and PASI 100 responses were decreased in patients with moderate-to-severe plaque psoriasis with guselkumab treatment interruption compared to those without interruption (9). Moreover, achievement rates for PASI 90 response at W56 were lower in patients with moderate-to-severe plaque psoriasis with bimekizumab treatment interruption than those without interruption (10). In line with these previous studies (8–10), the current study discovered that achievement rates for PASI 75, PASI 90, PASI 100, and sPGA 0/1 responses at W52 were lower in patients with moderate-to-severe plaque psoriasis with vunakizumab treatment interruption than those without interruption. Our findings revealed that vunakizumab treatment interruption had an adverse impact on skin clearance in patients with moderate-to-severe plaque psoriasis.

PROs are essential in assessing the efficacy of treatments, which encompass various dimensions of patients’ perceptions, including quality of life, symptoms, physical function, and mental health (23–25). The effect of biological treatment interruption on PROs in patients with moderate-to-severe plaque psoriasis has been investigated in some previous studies (9, 26). For instance, a previous study reported that tofacitinib treatment interruption was related to worse quality of life and pruritus, as evidenced by increased Itch Severity Item scores and DLQI scores in patients with moderate-to-severe plaque psoriasis (26). Another study claimed that improvements in DLQI score and Psoriasis Symptoms and Signs Diary symptoms or sign scores were smaller in patients with moderate-to-severe plaque psoriasis with guselkumab treatment interruption than those without interruption (9). In accordance with the findings of these previous studies (9, 26), we also discovered that PROs were worse in patients with moderate-to-severe plaque psoriasis with vunakizumab treatment interruption compared to those without interruption, as evidenced by increased DLQI score and I-NRS score, as well as decreased EQ-5D VAS score and SF-36 MCS. Our findings indicated that vunakizumab treatment interruption might impair quality of life, worsen pruritus symptoms, and decrease mental health in patients with moderate-to-severe plaque psoriasis.

Biologics show tolerable safety profiles for the treatment of moderate-to-severe plaque psoriasis (27–29). However, rare studies explored the impact of treatment interruption on the safety profiles of biologics in patients with moderate-to-severe plaque psoriasis. In this study, we found that the incidence of any adverse events (88.2% vs. 87.4%) did not differ between patients with vunakizumab treatment interruption and those without interruption. Nevertheless, analysis of individual adverse events revealed that the incidence of some specific events was different between patients with and without vunakizumab interruption, including hyperuricemia, hyperlipidemia, and elevated blood glucose. The potential reason might be that, in this study, patients were interrupted vunakizumab due to the COVID-19 pandemic, adverse events, and others; the interruption of vunakizumab affected the drug exposure, leading to the different incidence of these specific adverse events.

Subgroup analyses for treatment responses, PROs, and adverse events based on the times of vunakizumab treatment interruption in patients with moderate-to-severe plaque psoriasis were further conducted. Regarding efficacy, the increased frequency of vunakizumab treatment interruption was related to poor treatment response and PROs in patients with moderate-to-severe plaque psoriasis. Regarding safety, the incidence of any adverse events was not affected, but the incidence of some detailed adverse events was influenced by different frequencies of vunakizumab treatment interruption in patients with moderate-to-severe plaque psoriasis. Therefore, patients with moderate-to-severe plaque psoriasis should decrease the frequency of vunakizumab treatment interruption.

We observed that COVID-19 was the major reason for treatment interruption in patients with moderate-to-severe plaque psoriasis receiving vunakizumab. This finding was in line with a previous study (30). Based on this information, we speculated that during the COVID-19 pandemic, due to the safety considerations regarding the use of biologics (i.e., patients’ ability to fight infection), vunakizumab was interrupted, which led to less drug exposure over time, ultimately impairing the efficacy of vunakizumab in patients with treatment interruption.

This study contained several limitations. (1) The follow-up period was 52 weeks in the phase 3 trial. Considering that patients with moderate-to-severe plaque psoriasis require lifelong treatment, studies with long-term follow-up duration should be performed to validate the effect of treatment interruption on the efficacy and safety of vunakizumab in patients with moderate-to-severe plaque psoriasis. (2) Baseline features, including family history of psoriasis, hyperlipemia, PASI score, sPGA score, and SF-36 PCS, were unbalanced between the two groups, which might affect the findings of this study. (3) The generalizability of our findings should be further validated due to various reasons, such as strict patient selection in the phase 3 trial and the study region. (4) Further study could consider applying another grouping method, such as categorizing patients into ≤2, 3, 4, and ≥5 interruptions, to provide a deeper understanding of the impact of drug interruption frequencies on the efficacy and safety of vunakizumab.

In conclusion, vunakizumab treatment interruption contributes to poor treatment responses and quality of life in patients with moderate-to-severe plaque psoriasis. In clinical practice, the decision to interrupt vunakizumab treatment should be carefully deliberated, and patients with moderate-to-severe plaque psoriasis should be encouraged to maintain vunakizumab treatment.

## Data Availability

The original contributions presented in the study are included in the article/**Supplementary Material**, further inquiries can be directed to the corresponding author/s.
