# Supplementary material for: Impact of treatment interruption on the efficacy and safety of vunakizumab in patients with moderate-to-severe plaque psoriasis: a post-hoc analysis of a phase 3 trial
Source: Front Immunol. 2026 Jan 16;16:1639049. doi: 10.3389/fimmu.2025.1639049 (PMC12855095; doi:10.3389/fimmu.2025.1639049)
Supplement: Supplementary file 1 [file Table1.docx]

***Supplementary Materials***

**Supplementary Table 1.** Comparison of long-term response between patients with short and lengthy drug exposure.

| Items | Drug exposure | | *P* value |
| --- | --- | --- | --- |
|  | Short  (<median duration) | Lengthy  (≥median duration) |  |
| PASI 75 response, n (%) |  |  | 0.001 |
| No | 37 (17.5) | 19 (7.7) |  |
| Yes | 175 (82.5) | 229 (92.3) |  |
| PASI 90 response, n (%) |  |  | <0.001 |
| No | 55 (25.9) | 32 (12.9) |  |
| Yes | 157 (74.1) | 216 (87.1) |  |
| PASI 100 response, n (%) |  |  | 0.019 |
| No | 90 (42.5) | 79 (31.9) |  |
| Yes | 122 (57.5) | 169 (68.1) |  |
| sPGA 0/1 response, n (%) |  |  | <0.001 |
| No | 65 (30.7) | 33 (13.3) |  |
| Yes | 147 (69.3) | 215 (86.7) |  |

The median duration of drug exposure was 364 days. PASI, psoriasis area and severity index; sPGA, static physician's global assessment.

**Supplementary Table 2.** Comparison of adverse events among patients with different times of treatment interruption.

| Events, n (%) | Times of treatment interruption | | | | *P* value |
| --- | --- | --- | --- | --- | --- |
|  | 0 (n = 237) | 1 (n = 62) | 2 (n = 67) | ≥3 (n = 94) |  |
| Any | 209 (88.2) | 51 (82.3) | 58 (86.6) | 86 (91.5) | 0.376 |
| Hyperuricemia | 57 (24.1) | 8 (12.9) | 9 (13.4) | 13 (13.8) | 0.045 |
| URTI | 47 (19.8) | 16 (25.8) | 20 (29.9) | 18 (19.1) | 0.258 |
| Hyperlipidemia | 39 (16.5) | 13 (21.0) | 5 (7.5) | 4 (4.3) | 0.003 |
| Injection site reaction | 23 (9.7) | 9 (14.5) | 9 (13.4) | 9 (9.6) | 0.615 |
| Elevated ALT | 20 (8.4) | 8 (12.9) | 11 (16.4) | 12 (12.8) | 0.252 |
| Eczema | 17 (7.2) | 1 (1.6) | 2 (3.0) | 7 (7.4) | 0.263 |
| Elevated blood bilirubin | 16 (6.8) | 3 (4.8) | 5 (7.5) | 3 (3.2) | 0.594 |
| Pruritus | 15 (6.3) | 4 (6.5) | 0 (0.0) | 7 (7.4) | 0.105 |
| Urticaria | 11 (4.6) | 5 (8.1) | 5 (7.5) | 7 (7.4) | 0.514 |
| Elevated AST | 10 (4.2) | 0 (0.0) | 6 (9.0) | 8 (8.5) | 0.032 |
| Elevated blood glucose | 8 (3.4) | 5 (8.1) | 12 (17.9) | 15 (16.0) | <0.001 |

URTI, upper respiratory tract infection; ALT, alanine aminotransferase; AST, aspartate aminotransferase.

**Supplementary Table 3.** ANCOVA or multivariate logistic regression analysis on outcomes (Treatment interruption vs. Continuous treatment).

| Items | *P* value | OR/β | LL of 95% CI | UL of 95% CI |
| --- | --- | --- | --- | --- |
| **Long-term responses at W52** |  |  |  |  |
| PASI 75 response | <0.001 | 0.074 | 0.028 | 0.195 |
| PASI 90 response | <0.001 | 0.127 | 0.065 | 0.245 |
| PASI 100 response | <0.001 | 0.302 | 0.194 | 0.470 |
| sPGA 0/1 response | <0.001 | 0.121 | 0.064 | 0.226 |
| **PROs at W52** |  |  |  |  |
| DLQI score | 0.090 | 0.517 | -0.081 | 1.115 |
| DLQI 0/1 response | 0.601 | 0.870 | 0.516 | 1.467 |
| I-NRS score | 0.026 | 0.331 | 0.040 | 0.623 |
| EQ-5D utility index | 0.433 | -0.004 | -0.014 | 0.006 |
| EQ-5D VAS | 0.222 | -0.969 | -2.525 | 0.587 |
| SF-36 MCS | 0.064 | -1.196 | -2.463 | 0.071 |
| SF-36 PCS | 0.496 | 0.333 | -0.628 | 1.294 |
| **Adverse events** |  |  |  |  |
| Any | 0.197 | 0.666 | 0.359 | 1.235 |

ANCOVA, analysis of covariance; OR, odds ratio; LL, lower limit; CI, confidence interval; UL, upper limit; W52, the 52nd week after treatment initiation; PASI, psoriasis area and severity index; sPGA, static physician's global assessment; PROs, patient-reported outcomes; DLQI, dermatology life quality index; I-NRS, itch numerical rating scale; EQ-5D VAS, EuroQol-5D and visual analogic scale; SF-36, short form-36; MCS, mental component score; PCS, physical component score.

**Supplementary Table 4.** Reasons for treatment interruption.

| Reasons | Times of treatment interruption | | |
| --- | --- | --- | --- |
|  | 1 (n = 62) | 2 (n = 67) | ≥3 (n = 94) |
| Adverse events, n (%) | 4 (6.5) | 7 (10.4) | 23 (24.5) |
| COVID-19 epidemic, n (%) | 56 (90.3) | 63 (94.0) | 79 (84.0) |
| Others, n (%) | 7 (11.3) | 15 (22.4) | 15 (16.0) |

COVID, Coronavirus Disease.
